# Renin Angiotensin System, COVID-19 and Male Fertility: Any Risk for Conceiving?

**DOI:** 10.3390/microorganisms8101492

**Published:** 2020-09-28

**Authors:** Lorella Pascolo, Gabriella Zito, Luisa Zupin, Stefania Luppi, Elena Giolo, Monica Martinelli, Daniela De Rocco, Sergio Crovella, Giuseppe Ricci

**Affiliations:** 1 Institute for Maternal and Child Health, IRCCS Burlo Garofolo, 34137 Trieste, Italy; stefania.luppi@burlo.trieste.it (S.L.); elena.giolo@burlo.trieste.it (E.G.); monica.martinelli@burlo.trieste.it (M.M.); daniela.derocco@burlo.trieste.it (D.D.R.); sergio.crovella@burlo.trieste.it (S.C.); giuseppe.ricci@burlo.trieste.it (G.R.); 2 Department of Medicine, Surgery and Health Sciences, University of Trieste, 34137 Trieste, Italy

**Keywords:** SARS-CoV-2, male fertility

## Abstract

The current knowledge concerning the connection between severe acute respiratory syndrome coronavirus 2 (SARS-CoV-2) and the renin–angiotensin system (RAS) system in the male reproductive apparatus is still limited, so dedicated studies are urgently required. Concerns about the male fertility consequences of SARS-CoV-2 infection have started to emerge, since epidemiologic studies observed that this coronavirus affects male patients more frequently and with increased severity, possibly because of the hormone-regulated expression of angiotensin-converting enzyme 2 (ACE2) receptor. A disturbance in fertility is also expected based on studies of the previous SARS-CoV infection, which targets the same ACE2 receptor when entering the host cells. In addition, bioinformatics analyses reveal the abundant expression of ACE2 receptor in the male reproductive tissues, particularly in the testis. It has been proposed that pharmacological intervention favoring the angiotensin-(1–7)/ACE2/Mas receptor pathway and increasing ACE2 expression and activity could greatly prevent inflammatory lesions in this area. Finally, in laboratories performing assisted reproductive technologies it is recommended that more attention should be paid not only to sperm quality but also to safety aspects. Data about the potential infectivity of seminal fluid are in fact conflicting and do not exclude risks for both personnel and patients. The potential infectivity of SARS-CoV-2 in reproductive male tissues should be strongly considered and further investigated for the proper management of in vitro fertilization procedures.

## 1. Introduction: SARS-CoV-2 and COVID-19 Pandemic

On 31 December 2019, 27 cases of pneumonia of unknown etiology were reported in Wuhan (Hubei province, China). All these cases were found to be linked with the Wuhan wet market and were thereafter diagnosed as a novel coronavirus, named severe acute respiratory syndrome coronavirus 2 (SARS-CoV-2). Due to the human-to-human transmission with a reproduction number (R0) ranging from 2.24 to 3.58 [1], and the global expansion of the infection, the 2019 coronavirus disease (COVID-19) was declared a pandemic by the World Health Organization (WHO) on 11 March 2020. As of 8 September 2020, the number of cases has reached over 27,205,275 worldwide, with more than 890,392 deaths. USA, with 6,222,974 cases; India, with 4,280,422 and Brazil, with 4,137,521, are the most affected countries. In Europe, Spain (498,989), the UK (347,156) France (307,476) and Italy (277,634) are the states with the highest number of infections [2].

Coronaviruses are a family of single-strained, RNA-enveloped viruses causing enzootic infections in birds and mammals that in the last decades become capable of infecting humans as well. Human infection with these viruses often originates from a spill-over event from animals (original hosts) through intermediate hosts (reservoirs), after a sequential chain of genetic mutations [3]. Due to their characteristic virulence and their zoonotic origin, they are likely to produce severe outbreaks. Indeed, in 2002 the human SARS-CoV emerged from China, causing a deadly epidemic, whereas in 2012 the Middle East respiratory syndrome coronavirus (MERS-CoV) was identified.

SARS-CoV-2 has four structural proteins, namely the spike (S), envelope (E), membrane (M) and nucleocapsid (N) proteins. The surface unit (S1) glycoprotein is the most important feature for infection, since it is responsible for viral tropism, binding angiotensin-converting enzyme 2 (ACE2) receptors [4]. To achieve viral invasion, the S protein has to be primed by a cellular protease, the transmembrane protease serine 2 (TMPRSS2), that cleaves at the S1/S2 and S2′ sites. Then, the S2 subunit enables viral fusion with the host membrane, allowing the entry of viral particles through endocytosis [5,6]. ACE2 and TMPRSS2 are the two primary host molecules identified for the infectivity of SARS-CoV-2, although other possibly involved actors are under study [7]. It has been observed in vitro that other transmembrane serine proteases, namely TMPRSS4, 11A, 11D and 11E, promote SARS-CoV-2 (pseudovirions) S-protein-mediated fusion with the cellular membrane [8], as well as the proprotein convertase furin, which probably regulates virus pre-activation [9,10]. The mechanism of entry of SARS-CoV-2 is the same as in severe acute respiratory syndrome (SARS), however, the affinity of SARS-CoV-2 for ACE2 is 10–20-fold higher than that of SARS-CoV, which could explain its higher R0 [5,11].

SARS-CoV-2 infection usually manifests with fever, cough, fatigue, shortness of breath, sputum production, headache and myalgia. Gastrointestinal symptoms, hypogeusia and anosmia are also frequent. Most commonly, SARS-CoV-2 causes a mild flu-like disease, evolving to critical illness in the form of acute distress respiratory syndrome (ARDS), cytokine storm syndrome and death in predisposed people [12].

At the pulmonary level, it has been proposed that SARS-CoV-2 infection produces local downregulation of the ACE2 receptors, leading to a predominance of the ACE/angiotensin (Ang) II/Ang II type 1 receptor (AT1R) system over the ACE2/Ang 1–7/Mas receptor (MAS) system. This results in AT1R activation, inflammatory response in the targeted tissue and direct parenchymal injury [13].

Although the classical manifestation of COVID-19 is related to the respiratory system, SARS-CoV-2 causes a systemic disease with a massive inflammatory and immune response of the organism, leading to unpredictable and deleterious effects on other districts such as the liver [14], kidney [15] and cardiovascular system [16], as wells as the spleen, lymph nodes, brain, testis and skin [17]. All affected organs and tissues have high levels of ACE2 and TMPRSS2, as reported in online databases [18].

Numerous intravascular micro-thrombotic events are frequently found during autoptic investigation, suggesting a hypercoagulative status in severe COVID-19 patients [19].

SARS-CoV-2 transmission mainly occurs through respiratory droplets and fomites, but the virus can also survive on surfaces [20]. SARS-CoV-2 has been also isolated from blood and stools, raising questions about viral shedding and transmission through other body fluids, including semen [21]. It has been calculated that asymptomatic infections may account for as much as 45% of all COVID-19 cases, playing a significant role in the early and ongoing spread of SARS-CoV-2 infection [22].

COVID-19 predominantly affects adults and old people. Moreover, it affects a slightly higher percentage of male patients [23], as well as displaying more severe disease and a higher mortality rate (especially in the 60–90 age range) in males compared to females [24].

This could suggest a gender predisposition to infection, although this ratio varies amid the published articles and an explanation of this difference has still to be elucidated [25,26]. Several observations could explain these gender differences. ACE2 is expressed more in women than in men, perhaps due to an estrogen-dependent regulation [27] and a differences in *ACE2* promoter methylation between genders [28]. In fact, the *ACE2* gene is in the Xp22.2, a chromosomal region that could escape X inactivation [29]. On the other hand, the TMPRSS2 concentration is higher in men than women, through an androgen-dependent gene expression [30]. Moreover, since in vitro studies demonstrated that estrogens can overrule oxidative and inflammatory stress, it has been hypothesized that the upregulation of ACE2 in women could also be protective against tissue damage [31,32].

It can be suggested that ACE2 acts as a double-edged sword. Indeed, while low ACE2 levels may decrease the probability of infection, on the other hand, poor availability of ACE2 for physiological host homeostasis could lead to a more severe disease. Moreover, higher levels of TMPRSS2 and increased activity of the protease in men could promote viral invasion [33].

The gender differences observed raise questions about the possible repercussions that SARS-CoV-2 could have on the male reproductive system, but few studies have been conducted and most of them have been underpowered and uncertain [34]. Since ACE2 and TMPRSS2 are expressed in the male reproductive tissues, they are potential targets of SARS-CoV-2, suggesting that the infection may have an effect on male fertility.

Genomic susceptibility towards COVID-19 development has been postulated. The research is mainly focused on the *ACE2* gene. A preliminary study by Cao and colleagues [35], searching for genetic polymorphisms of *ACE2* in different populations, found no evidence to support different susceptibility according to *ACE2* variants. Instead, Hussain et al. [36] identified two *ACE2* alleles presenting a low binding affinity with the SARS-CoV-2 spike protein, indicating a possible resistance to infection. In addition, Darbani [37] speculated about the possible influence of some variants in *ACE2* in its interaction with the viral S1 protein and consequently on the viral infectivity.

Lately, a possible genetic component in the severity of COVID-19 among the Italian population was ascribed to particular *TMPRSS2* variants, which may influence viral infection [38].

Recently, Lopera and colleagues [39] investigated the role of genetic variants in *ACE2* and *TMPRSS2* genes. They examined 178 quantitative phenotypes and 58 medications, but no significant evidence that common and low frequency variants are related to COVID-19 susceptibility or severity was discovered. They observed only the association of some single-nucleotide polymorphisms (SNPs) within the *ACE2* gene with the use of non-steroid anti-inflammatory and antirheumatic drugs (NSAIDs) and with the employment of angiotensin II receptor blockers (ARBs) in combination with other therapies, but other studies and a large and well characterized cohort are necessary to confirm these data and to research new genetic associations.

The aim of this review is to summarize and comment on the principal concepts and evidence regarding the possible impact of SARS-CoV-2 infection on the male reproductive system and therefore on fertility. Specifically, we focus on the local components of the renin–angiotensin system (RAS) that may be negatively influenced by SARS-CoV-2 infection, namely, ACE, ACE2, AT1R, AT2R, MAS and the protease TMPRSS2, necessary for virus entry. Finally, we discuss the safety aspects for laboratories performing assisted reproductive technologies, and the importance of carefully monitoring sperm quality and male fertility in the COVID-19 pandemic era.

## 2. Importance of the Renin–Angiotensin System (RAS) in Male Fertility

The RAS is a complex enzyme-protein cascade which, through the generation of cellular mediators, regulates renal function. It plays a leading role in the control of blood pressure and vascular tone, as well as in hydro-saline homeostasis, affecting the volume of plasma and other fluids. Renin is a proteolytic enzyme that is accumulated in the juxtaglomerular cells of the kidney and released in response to various stimuli such as a reduction in blood pressure or sodium concentration, or beta activation [40]. Upon activation, renin cleaves its substrate, angiotensinogen (AGT), to produce Ang I. Ang I conversion to Ang II (octapeptide) is mediated by endothelial ACE, primarily in the lung endothelium.

Ang II binds predominantly to its AT1R receptors in the kidney, adrenal cortex, arterioles and brain, having a subsequent systemic effect due to vasoconstriction and the release of aldosterone from the adrenal gland, resulting in sodium reabsorption and increased blood pressure [41]. Ang II is the main, but not unique, effector of the RAS [42]. Indeed, Ang II can be processed by ACE2, producing Ang 1–7, which binds MAS; moreover, Ang I can be converted in Ang 1–9 by ACE and then in Ang 1–7 by ACE2; whereas AT2R is a low-abundance receptor for Ang II that reinforces the Ang 1–7 cascade.

**Figure 1:**
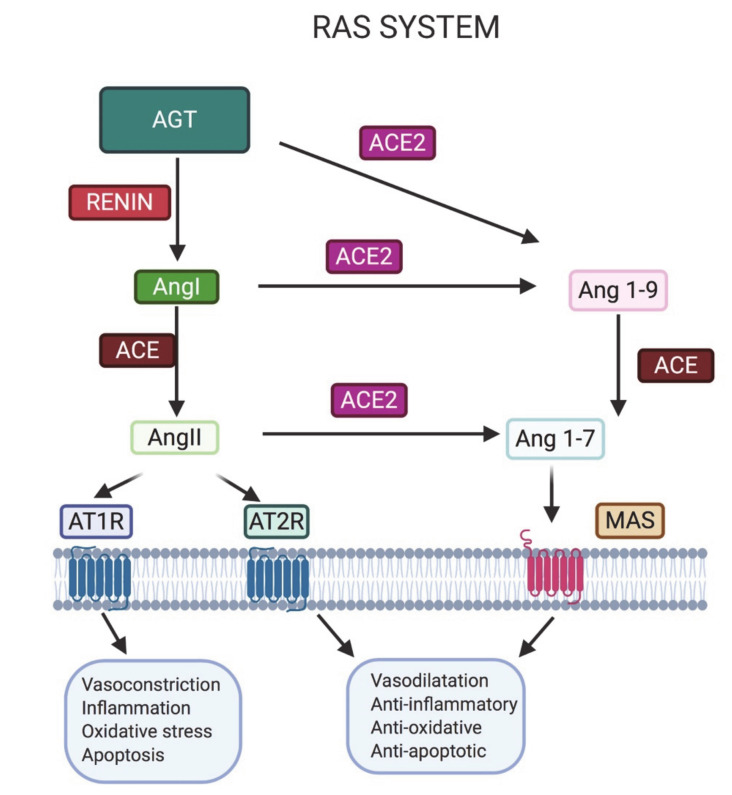
The renin–angiotensin (AGT) system (RAS): The angiotensin converting enzyme (ACE) enzyme is responsible for the production of Angiotensin (Ang) II, whereas ACE2 is responsible for the production of Ang 1–7 starting from the same precursor (Ang I) or from degradation of Ang II. There is a balance between Ang II versus Ang 1–7 because they mediate opposite actions. Finally, there is a balance between receptors because Ang II type 1 receptor (AT1R) mediates opposite actions to AT2R and Mas receptor (MAS).

As shown in Figure 1, the RAS is a complex mechanism with a number of elements that mediate various actions that counterbalance each other: one being vasoconstrictor/proliferative in which Ang II (axis ACE/Ang II/AT1R) plays the main role, and the other being a vasodilator/anti-proliferative action, fof which the major effector is Ang 1–7 (axis ACE2/Ang 1–7/MAS).

Renin and all other components of the system can be synthesized, in a carefully regulated way, in numerous tissues, together with tissue specific proteins and receptors [42]. Indeed, ACE is expressed by a variety of sites, including the epithelial cells of the proximal tubules of the kidney, vascular endothelium and blood, adrenal gland, heart, adipose tissue, gonads, placenta, liver, intestinal brush border and, possibly, the brain [43,44,45]. Beyond the classical view of the endocrine RAS pathway, several local RAS have been described that have different functions and act independently of each other and of the circulating RAS [46].

The role of a local RAS in the male reproductive system has been demonstrated, although its relevance to fertility needs further investigation [47]. The male human reproductive tract and the testes express all components of the RAS pathway [48,49,50,51,52], including ACE2 producing Ang 1–7 and its receptor MAS [52,53,54]. The male reproductive system is isolated from the plasmatic RAS by the blood–testicular barrier. This supports the evidence for the local synthesis of RAS components in the epididymis, prostate, testes, seminal fluid and spermatozoa, as shown in Table 1 [55]. Renin is supposed to be produced in the Leydig cells, where Ang I and Ang II have also been detected [47].

Notably, the testicular RAS seems to be relevant in modulating sperm fertilization ability [55,56].

Testicular renin, ACE, AGT and total ATR increase with plasma gonadotropins at the onset of puberty, thus suggesting a role in pubertal development. ACE1 and ACE2 have gained recognition as significant regulators of the physiology and pathology of the reproductive system: ACE is deemed to be involved in sperm capacitation; and ACE2 is thought to be involved in the regulation of spermatogenesis [54].

### 2.1. ACE2 in the Male Reproductive System

ACE2, a dipeptidyl carboxydipeptidase, is a type I integral membrane glycoprotein of 805 amino acids with a single catalytic domain and its functional activity is that of a typical zinc metallopeptidase. In the RAS, ACE2 degrades angiotensin II into Ang (1–7). The former has potent effects on vasoconstriction, inflammation and fibrosis, whereas the latter has opposite effects, like vasodilatation, anti-proliferation and apoptosis [69].

Several authors provided a comprehensive summary of ACE2 expression levels across all major human tissues, based on public data from multiple previous studies conducted on mRNA, single-cell RNA and protein level.

In physiological conditions, ACE2 is expressed in the heart, kidneys, lungs, liver, intestine and brain datasets, but also in the testes and placenta, as confirmed by immunohistochemical and scRNA-seq datasets [59,70,71,72,73,74]. Despite its extensive mRNA presence in different tissues, recent studies using ACE2 protein expression profiles and transcriptomic datasets could confirm its presence only in the bowel, renal proximal tubules, gallbladder, testicular Sertoli and Leydig cells, and in cardiomyocytes. In lungs and pneumocytes the expression of ACE2 appears to be limited. Indeed, the role of ACE2 in viral infection and correlation to clinical condition is still to be unraveled [70].

**Figure 2:**
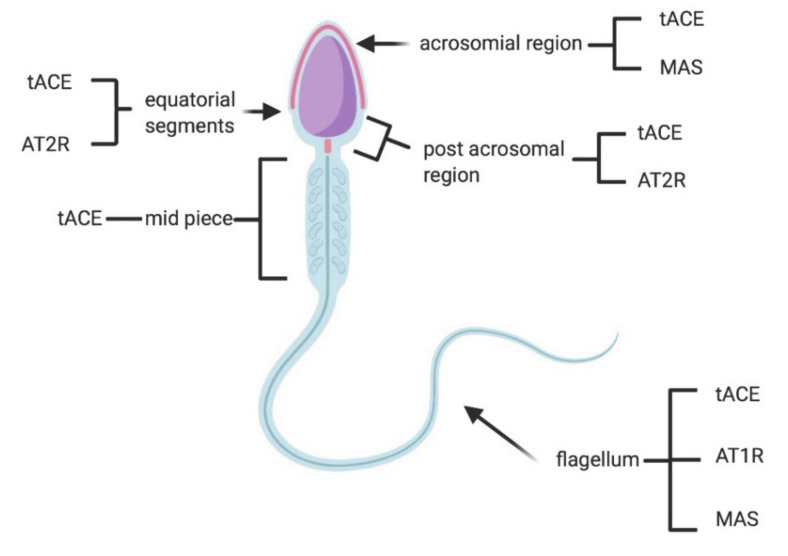
Expression of RAS components in different localizations throughout spermatozoa. tACE: testicular angiotensin converting enzyme; AT1R: angiotensin II type 1 receptor; AT2R: angiotensin II type 2 receptor; MAS: Mas receptor.

Regarding the human male reproductive system, Fan et al. showed that ACE2 protein and mRNA expression in the testes is almost the highest in the body, especially in Leydig cells and seminiferous ducts [59]. Similarly, Zhang J et al. found high levels of ACE2 mRNA and protein expression in the testes and spermatids [73]. Hikmet et al. confirmed the findings of previous studies with cell-type-specific localization of ACE2 based on immunohistochemistry, showing ACE2 expression to be mainly localized in testicular Sertoli cells, Leydig cells and glandular cells of seminal vesicles [70]. Wang and Xu found that ACE2 is namely present in spermatogonia, Leydig and Sertoli cells [67] (Figure 2, Table 1).

The observation that infertile men with severe spermatogenesis impairment have decreased ACE2 levels is the strongest evidence that ACE2 receptors affect fertility [53]. The receptor has been recognized as an important regulator of steroidogenesis, epididymal contractility and sperm cell function [54].

Different factors, including salty diet, aging, as well as comorbidities, e.g., diabetes, pulmonary, renal, cardiovascular, and coronal heart diseases, may impair ACE2 production and activity, [72]. For instance, it was demonstrated that a diet rich in sugars and fats causes a downregulation of ACE2 [75], therefore suggesting that obese subjects have a lower expression of ACE2.

The fact that obese male subjects often suffer hypogonadism [76] could be explained by the lower testicular expression and/or activity of ACE2 in Leydig cells, leading to an imbalance of the RAS cascade, thus favoring the Ang II/AT1R pathway. This could provoke inflammation and alteration of the physiological function of this type of testicular cells, generating a reduction of TSH production and consequent hypogonadism.

Notably, nicotine can also influence the RAS, increasing the expression and/or activity of renin, ACE and AT1R and downregulating the expression and/or activity of ACE2 and AT2R [77]. Cigarette smoking can also inhibit the antioxidant system of the testes and increase reactive oxygen species (ROS) numbers, promoting DNA fragmentation, cell apoptosis and general impairment of sperm function, leading to reduced semen quality, reproductive hormone system disorders and consecutively lower male fertility [78]. It is possible to speculate that the detrimental effects of tobacco on male fertility are due to a nicotine-induced imbalance of the RAS system, resulting in a lower protective activity of ACE2 on the testes.

### 2.2. ACE

ACE exists in two isoforms: somatic (sACE) and testicular (tACE). In the male reproductive system, sACE is present in seminal plasma and on the surface of the epididymal epithelium, Leydig cells and prostate [57], whereas tACE is exclusively found in male germinal cells, specifically in the Golgi apparatus of spermatids at different stages of spermiogenesis and on the plasma membrane of the acrosomal region, equatorial segment, post acrosomal region, mid-piece and the flagellum of ejaculated spermatozoa [58,79].

Remarkably, sperm motility seems to be negatively correlated with both the percentage of tACE-positive spermatozoa and the number of tACE molecules per spermatozoon, since tACE inactivaes bradykinin [80], a stimulator of sperm movement [81]. Absent or aberrant expression of human tACE protein in spermatozoa causes low rates or failure of fertilization [82] In addition, an association between tACE and embryonic quality has also been found [83]. It is intriguing that semen samples with a high percentage of tACE-positive spermatozoa, together with a low number of tACE molecules on the surface of the spermatozoa, are associated with better embryonic quality [84].

To the best of our knowledge, no data are available regarding the relationship between tACE and ACE2.

### 2.3. AT1R and AT2R

AT1R and AT2R are G-protein-linked receptors [85], and are the targets of Ang II [56].

AT2R binds with Ang II with less affinity than AT1R. Recent evidence has shown that signals mediated by AT2R offset the effects induced by AT1R activation, resulting in differentiation, inhibition of proliferation and apoptosis [86].

**Figure 3:**
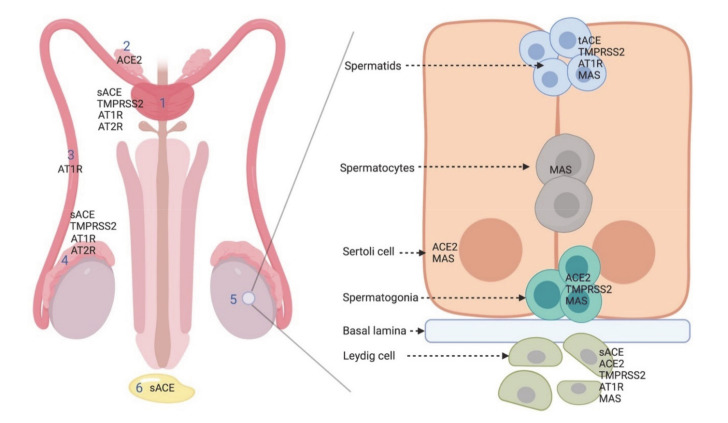
On the left: localization of renin–angiotensin system (RAS) components in human male reproductive system (1, prostate; 2, vas deferens; 3, epididymis; 4, seminal plasma). On the right: RAS components at the cellular level in spermatidis, spermatocytes, Sertoli cells, spermatogonia, basal lamina, Leydig cell. (ACE: angiotensin-converting enzyme; sACE: somatic ACE; tACE: testicular ACE; TMPRRS2: transmembrane protease serine 2; AT1R: angiotensin II type 1 receptor; AT2R: angiotensin II type 2 receptor; MAS: Mas receptor).

AT2R is present in the prostate, epididymis and in human semen, where it is mainly located at the equatorial segment of the head and in the post-acrosomal area of spermatozoa [55,61,62]. AT2R levels seem to be positively correlated with sperm concentration and progressive motility, suggesting that this molecule may be involved in the control of sperm movement and it could be considered an important indicator of sperm quality [62]. AT1R is detected in developing spermatids and in the tail of mature spermatozoa [60,87,88,89,90], but also in Leydig cells, the epididymis, vas deferens and prostate [55,61,87,91,92,93,94,95,96,97]. AT1R is involved in sperm function and fertility, such as maturation, capacitation and acrosome reaction, steroidogenesis and epididymal contractility [54,55,68] (Figure 2 and Figure 3; Table 1).

### 2.4. MAS

MAS receptor is a G-protein-coupled receptor which binds Ang 1–7, produced by ACE2, and once activated opposes or supports many of the effects deriving from activation of AT1R and AT2R, respectively [98,99,100].

Ang 1–7 has been detected in the cytoplasm of Leydig cells and in external layers of the seminiferous tubules, particularly in the cytoplasm of Sertoli cells and primary spermatocytes. 

The MAS receptor has been also localized in the cytoplasm of Leydig cells and in the seminiferous epithelium, from Sertoli cells and spermatogonia to spermatozoa (Figure 2 and Figure 3; Table 1) [53]. MAS*^−^*^/*−*^ mice are fertile [101], with a number of Sertoli and Leydig cells comparable to those of wild-type animals. However, they show a significant reduction in testis weight, and an augmentation of apoptotic cells during meiosi, and of giant cells and vacuoles in the testis, resulting in a decrease of daily sperm production [102]. In agreement with these results, Reis et al. [53] observed that men with severely impaired spermatogenesis had lower levels of ACE2 and MAS mRNAs and no protein expression (detected by immunohistochemistry) of Ang 1–7 and MAS in seminiferous tubules, in comparison to fertile subjects. Valdivia et al. [63] reported the presence of the functional MAS receptor in human spermatozoa, especially in the sperm head, over the acrosomal region and in the flagellum, where it participates in the regulation of sperm motility in healthy and astenozoospermic subjects (Figure 3).

### 2.5. Drugs Acting on RAS System

Among the drugs that target RAS receptors, there are the ACE inhibitors (ACEIs) and the antagonists of Ang II receptors (AT1R and AT2R), both of which are able to reduce RAS function and are consequently used for their antihypertensive activity. It remains unclear whether RAS inhibitors such as ACEIs and angiotensin II type 1 receptor blockers are associated with improved or worse clinical outcomes during COVID-19 [103]. Although RAS blockade is suggested to possibly increase ACE2 expression, there is no evidence that RAS inhibitors are dangerous during SARS-CoV-2 infection; on the contrary, they seem to be beneficial in animal studies [103]. This topic is still a matter of debate.

Since Ang II inhibits Leydig cell function and testosterone production, ACEIs have become first-line therapy for the management of idiopathic oligozoospermia, although some controversial results have been observed [54]. Some ACEIs, such as lisinopril, could exert positive effects on sperm count, motility and morphology by blocking the conversion of bradykinin into inactive peptides [104,105], thus enhancing Sertoli cell function and spermatogenesis [106]. On the contrary, Saha et al. did not evidence any improvement in sperm quality and quantity after treatment with lisinopril [107].

Some experimental molecules like xanthenone and diminazene aceturate, antiparasitic drugs, have been used in vivo on animal models as ACE2 activators and seem to possess antifibrotic and anti-inflammatory properties [108], thus representing a possible therapeutic option against SARS-CoV-2 infection in humans; however, no definitive findings report a clear benefit in COVID19 patients who used these drugs, mostly without controlled drug trials. Furthermore, it could be speculated that similar molecules may also act with beneficial effects at the reproductive system level.

## 3. TMPRSS2 in the Male Reproductive System: Another Actor in SARS-CoV-2 Infection

The entry of SARS-CoV-2 requiresTMPRSS2 [5,109], a member of the serine proteases family, one of the largest families of proteolytic enzymes, involved in many physiological processes, such as development, digestion, coagulation, inflammation, fertility and immunity—but it is also implicated in the activation of respiratory viruses [110,111,112,113,114,115]. TMPRSS2 is classified as a type II transmembrane serine protease (TTSP) characterized by an N-terminal transmembrane domain that anchors the protein to the plasma membrane and the catalytic serine protease domain at the C-terminal. TMPRSS2 is synthesized as zymogen and then activated by autocatalytic cleavage [116,117].

According to RNA and protein expression databases (Human Protein Atlas, FANTOM5 and GETx) [18,118,119] and the available literature, TMPRSS2 is localized in the epithelial cells and it is maximally expressed in the prostate gland (luminal epithelial cells). Only moderate or low level expression is found in other organs, including the kidney, colon, small intestine, pancreas, salivary gland, stomach and lungs [64,65,66,120,121]. TMPRSS2 expression in the testis is controversial (Table 1). Lin et al. [121] did not observe its expression in the testis, whereas Lucas et al. [65] reported TMPRSS2 expression in Leydig cells and in the epididymis. Using expression repositories (Human Protein Atlas, FANTOM5 and GETx) [18,118,119] low levels of TMPRSS2-RNA have been observed in testes, but TMPRSS2-protein was not detected by immunohistochemistry. Recent studies of scRNA-seq profiling indicate that TMPRSS2 expression is concentrated in spermatogonia and spermatids [67]. Other authors found secreted forms of TMPRSS2 in the human semen as components of prostasomes, suggesting its possible role in the regulation of sperm function [65,66]. *TMPRSS2^−^*^/*−*^ mice have a normal development and are fertile, with no alteration of their prostate and seminal vesicles. However, TMPRSS2 could have a different effect on humans [122]. TMPRSS2 protein is widely studied and known for its role in prostate cancer development; indeed, the transcription of the *TMPRSS2* gene is regulated by androgenic hormones and a deregulation of androgen signaling determines prostate cancer progression [64,65,117,121,123].

## 4. SARS-CoV-2 Detection in the Reproductive System and Effects on Fertility

The effects of SARS-CoV-2 on human male reproductive systems have not been fully elucidated, although many indications come from studies on previous coronavirus species.

Interestingly, SARS-CoV patients showed significant damage in reproductive organs. SARS-CoV was demonstrated to affect the testes directly through ACE2 receptor, which is highly expressed in the human reproductive system. The same authors identified SARS-CoV in the epithelial cells of testicular seminiferous tubules and Leydig cells through semi-quantitative analysis, in situ hybridization and immunohistochemistry, providing direct evidence of viral testicular damage [124].

Gu et al., in eight post-mortem SARS-CoV patients, observed testicular tissue with focal atrophy despite lacking identifiable SARS-CoV viral RNA [125], similarly to Ding et al., who also did not detect SARS-CoV RNA in the testes [126].

Testes of autopsied SARS-CoV patients displayed germ cell destruction, showing few or no spermatozoa in the seminiferous epithelium or lumen and a mixed cellular infiltrate, with a significantly increased number of apoptotic spermatogenic cells [127]. It has been proposed that leukocyte infiltration interferes with Leydig cells’ production of testosterone, damages the blood–testis barrier, and destroys the seminiferous epithelium directly [128]. On the other hand, orchitis may be induced by inflammatory cytokines activated by the autoimmune response with autoantibody development within the tubules [129,130].

A study by Xu et al. reported that persistent temperature rise, caused by the high fever during SARS-CoV infection, may have an indirect effect on testicular dysfunction [128]. It is well known that fever alone causes heat-induced spermatogenesis damage [131]. In addition, in these conditions the testis shows leukocyte infiltration and extensive IgG precipitation in interstitial tissue, which causes germ cell and Sertoli cell destruction, mild fibrosis and minimal Leydig cell hyperplasia, but exhibits extensive germ cell destruction with few or no spermatozoa in the seminiferous epithelium and the lumen [128].

Interestingly, the other coronavirus that affects the human beings [132], Middle East Respiratory Syndrome-CoV (MERS-CoV), requiring dipeptidyl peptidase 4 (DPP4) as a functional virus receptor [133] rather than ACE2, has no known effects on the reproductive system.

Therefore, it can be supposed that SARS-CoV-2 infections may affect male reproductive potential. Theoretically, testicular damage and subsequent infertility could be caused by SARS-CoV-2 by direct viral invasion through the binding of the virus to local ACE2 receptors or mediated by host immunological and inflammatory responses [134]. Data suggest that ACE2 also plays a role in spermatogenesis [54], thus implying a potential action of the virus on germinal cells.

Membrane expression and tissue activity of ACE2 seem to be key players in cells’ susceptibility to SARS-CoV-2 infection; thus, high expression could correlate with higher virus entry. On the other hand, ACE2 expression seems to be protective during inflammation (Figure 1), although its exact roles during the disease and its severity could be complex and require further investigation [73].

As a matter of fact, ACE2 is expressed, among others, in seminiferous duct cells, spermatogonia, Leydig cell and Sertoli cells of the adult testis. Notably, in the testis, ACE2-positive cells exhibit an abundance of transcripts potentially associated with viral replication and a lower number of transcripts associated with gametogenesis [135]. This provides evidence that human testes are potential target of SARS-CoV-2 infection, facilitating viral replication with consequent damage especially on gonadal hormone secretion and spermatogenesis [67]. Moreover, transmembrane serine protease 2 (TMPRSS2), which seems to enhance ACE2-mediated viral entry, is expressed in prostatic epithelial cells, in epididymal cells and in some cells of the germline (spermatogonia and spermatids). Consequently, the testis may be an organ at high-risk of potential infection [136]. Cell-type-specific expression patterns of genes encoding for viral entry proteins (ACE2 and TMPRSS2) are critical to identify tissues at risk, and their presence in reproductive system cells could also imply an impact on fertility. scRNA-seq analysis found high ACE2 expression in renal tubular cells and testicular cells, which suggested a potential mechanism of infection and direct damage of renal tubules and testes by SARS-CoV-2 [59]. However, the analysis did not consider the presence of TMPRSS2 and did not correlate gene expression to the clinical outcomes.

Other studies based on scRNA-seq data showed no co-expression of ACE2 and TMPRSS2 in any testicular cell type. The authors concluded that sperm cells should not be at risk of viral entry and that spreading the virus through intercourse and manipulation during assisted reproduction techniques is unlikely, although it is important to note that another putative receptor is present in this area, i.e., basigin (BSG/CD147), as well as another protease, cathepsin L (CTSL), that could cleave the S protein. Nevertheless, their involvement in viral entry is still to be proven [137]. Sertoli cells, which were previously deemed to be interested in SARS-CoV-related orchitis [128], were not investigated in that study.

A first Italian case report failed to identify SARS-CoV-2 RNA in the semen and urine of a man diagnosed with the virus in nose-pharyngeal swab [138]. Later, no evidence of the virus was found in the semen of men infected by COVID-19 at both acute and recovery phases [139,140,141] and in one post-mortem testis sample [139]. Similarly, in a mild COVID-19 patient cohort, no SARS-CoV-2 RNA was detected after a median of 32 days from the first symptoms; moreover, total sperm number, total motile sperm counts and sperm morphology parameters were all within normal ranges [142].

On the contrary, more recently a study on 38 semen samples from men already affected by COVID-19 and individuals recovering from the disease showed that SARS-CoV-2 can be present in the semen of both groups of patients. As this suggests that viral transmission through semen cannot be excluded, the study group concluded that abstinence or condom use might be considered as preventive measures for these patients [143].

The latest evidence showed no SARS-CoV-2 particles in 34 semen samples from Chinese men within 1 month since a COVID-19 diagnosis. Scrotal discomfort, indicative for orchitis, coexisted in six of the 34 patients. Nonetheless, single-cell transcriptome data from the same study group displayed low ACE2 RNA expression at this site, suggesting a potentially different pathogenic mechanism for SARS-CoV-2 in the testes, which does not include viral entry [144].

Notably, in a cohort of 11 COVID-19 patients, only one showed a RT-PCR positivity to SARS-CoV-2 in the testis after 30–40 days from the onset of the symptoms, but no cases presented viral particles in the testis specimens analyzed by electron microscopy. Nevertheless, the authors reported mild (two cases), moderate (five cases), or severe (four cases) morphologically damaging changes in the seminiferous tubules, characterized by the loss of tubular cell mass in the lumen, interstitial edema with infiltration of T-lymphocytes and histocytes, vacuolization and detachment of Sertoli cells from the basal membrane and the reduction of Leydig cells, although spermatogenesis was not affected. These novel findings raised the question of whether the damage observed may be linked to direct injury by a local infection with the SARS-CoV-2 S protein, or to different indirect factors such as fever or hypoxia, since the virus was not detected in the specimens. The authors suggested that although spermatogenesis was maintained at the time of analysis, the alteration in Sertoli and Leydig cells may lead to future impairment [145].

Moreover, in a Brazilian study on COVID-19 patients, orchitis was detected in two out of 10 fatal cases, confirming the male reproductive tissue repercussions of SARS-CoV-2 infection [17]. In addition, in a case report, a 43-year-old male patient with fatal COVID-19 pneumonia reported testicular pain. The computed tomography scan showed symmetrical enhancement of the testes, epididymis, testicular artery and pampiniform plexus, whereas the ultrasound scan registered a picture of epididymitis [146]. Similarly, testicular pain was also reported in in a 42-year-old man with SARS-CoV-2 infection with a benign clinical course [147].

In another study, sperm quality (sperm concentration, total number of sperm per ejaculate, total number of progressive motile, total number of complete motile) was assessed in affected, recovered and non-affected patients. A moderate infection was associated with a statistically significant impairment of sperm quality. This effect could be due to SARS-CoV-2 infection directly, depending on the severity of symptoms and a temporary high viral load during acute phase of the disease. Moreover, the potential effect of treatments (lopinavir/ritonavir or hydroxychloroquine) on sperm quality indicators cannot be excluded, although their effect is not clearly elucidated, and their use is restricted to a short period. As a drawback of that study, as the subjects provided just one sperm sample, nothing is known about the previous sperm parameters [148].

One retrospective study provided the first evidence regarding the alteration of sex-related hormones under COVID-19. After comparing follicle-stimulating hormone (FSH), luteinizing hormone (LH), testosterone (T), prolactin, anti-Mullerian hormone AMH and oestradiol (E_2_) levels in CoVID-19 and fertile control men, they found a significant increase of LH levels and a decreased T:LH ratio, suggesting an impairment in the steroidogenic function of the testis [149].

Notably, it is known that fever can impact spermatogenesis, thus male fertility may be decreased for 72–90 days following symptomatic COVID-19 due to lower sperm concentration and motility, although this association was never confirmed [131]. Moreover, the cytokine storm and inflammation associated with severe cases of COVID-19 might negatively impact spermatogenesis [150] and also transiently impair the blood–testis barrier [151]. Alternatively, it has also been proposed that micro-coagulation and vasculitis could account for the orchitis-like syndrome in COVID-19 [152], similarly to what has been reported in SARS-CoV [153].

Finally, it is important to consider that RAS homeostasis impairment caused by ACE2 subtraction through SARS-CoV-2 binding, as seen in the lungs [13], could also potentially have repercussions on the male reproductive system. Data suggest that the ACE2/Ang 1–7/MAS axis plays a role in spermatogenesis [54], although the putative mechanism of action of the virus on germinal cells is unknown. Reduction of the ACE2 available for physiological functions is likely to result in a downregulation of the ACE2/Ang 1–7/MAS axis, with an impairment in spermatogenesis as a consequence, as seen in infertile subjects [53]. To speculate, we could expect to observe a condition similar to that of MAS-knockout mice, with an aberrant expression of genes involved in mitochondrial function and testicular steroidogenesis [102]. However, better knowledge of the role of RAS in the reproductive system is required.

Indeed, the secretion of anions and fluid by the epididymal epithelium is controlled by RAS components and has strong impact on the functionality of this apparatus, affecting sperm maturation and expulsion [55]. Epididymal cells create a specialized luminal microenvironment fundamental to supporting sperm development, where the correct ionic concentration, characterized by a low level of Na^+^, Cl^−^ and HCO_3_^−^, is mandatory to regulate the acidification that contributes to keeping the spermatozoa in a quiescent state [154]. It would be interesting to unravel if and how this equilibrium is altered during infection.

## 5. Safety of Samples in PMA Procedures

During the current COVID-19 pandemic, non-essential treatments have been suspended or reduced to guarantee social distancing and restrict access to hospitals, which have to cope with overcrowding. Moreover, due to uncertainties regarding vertical transmission of the virus and effects on pregnancy, reproductive medicine associations worldwide have published recommendations and position papers about the management of candidates for fertility treatments.

In March 2020, the European Society of Human Reproduction and Embryology (ESHRE) advised people “to avoid becoming pregnant at this time”. For those patients already having treatment, they suggested “considering deferred pregnancy with oocyte or embryo freezing for later embryo transfer” [155]. The American Society for Reproductive Medicine (ASRM) also issued a statement suggesting suspending “non urgent” treatments and delaying embryo transfers if possible [156].

Resumption of fertility treatments is now underway in different countries, depending on their COVID-19 epidemiological situation. ESHRE and ASRM advise that treatment resumption is strictly bound to each center’s ability to limit the risk to patients, staff, physicians and other healthcare providers [156,157,158].

Telemedicine should be encouraged and when face-to-face consultation is needed, and appropriate precautions should be taken, such as routine sanitation, avoiding crowds and the use of personal protective equipment (PPE). Patients undergoing Assisted Reproductive Technology (ART) treatments and operators who are infected, who have had contact with positive or suspected people or who have symptoms must obviously be restricted from entering clinics [159].

According to ESHRE guidance for phase 2, a patient’s risk evaluation should be carried out 2 weeks prior to the start of the treatment, at the start of ovarian stimulation, at oocyte retrieval and before embryo transfer. Asymptomatic patients triaged as low risk (negative clinical history, lifestyle compatible with low/minimal risk of contact with potentially infected individuals) should undergo ART with no further COVID investigation. If they show any COVID-19-related symptom at any time during the cycle, serological tests for the detection of IgM and IgG antibodies and/or RT-PCR testing for COVID-19 should be performed. In the absence of antibodies, patients are eligible for starting the treatment. Positivity for IgG and IgM, meaning exposure to the virus and potential for transmission, imposes the suspension of the procedure and referral for further testing. Patients and/or partners who are symptomatic or COVID-19-positive should not undergo the treatment and should be referred for further testing and isolated [157].

No available evidence has shown the presence of the virus in follicular fluid [160]. Instead, data about the potential infectivity of seminal fluid are conflicting. Although the presence of SARS-CoV-2 virus in the seminal fluid of infected and hospitalized patients has been demonstrated, no conclusion about transmission can be derived and further studies are needed to confirm this hypothesis [143].

Due to the uncertainty about the safety of manipulating semen samples and the effects of semen contamination, laboratory activity should be reorganized in relation to risk management. During the COVID-19 pandemic, transmission-based precautions are essential to protect patients and operators from contagion by aerosolization or contact with contaminated surfaces [156]. Continuous inspection of tanks and generators, monitoring of liquid nitrogen (LN2) status daily, staggering of embryology staff and checking of gametes/embryos stored in vapor tanks and levels of LN2 should be carried out [161].

ESHRE advises professionals to strictly follow routine good laboratory practice and to take extra care to reduce exposure to follicular fluid and sperm, by dilution and safe disposal of fluids in individual closed containers, as quickly as possible [157].

During ART, the semen sample could be contaminated by contact with the outer surface of the collection container or by the operator, who manipulates the sample during all the stages of processing. Serological screening of operators and the use of sterile protective equipment such as masks and gloves minimize this risk. Moreover, the nitrogen used for cryopreservation of seminal fluid can be a source of contamination. It may be advisable to use sterile nitrogen to wash the device at the thawing/warming stages [162].

When processing semen in virus-positive patients, the viral titer should be kept at the lowest possible level. Special washing refers to an additional swim-up step for sperm from the pellet, which results from the density gradient [163].

ESHRE, in a document dated 2 April 2020, states that the repeated washing phases required by culture and freezing procedures probably minimize the risk of viral contamination of gametes and embryos in the in vitro fertilization (IVF) laboratory, resulting in a high dilution of any possible contaminants. High security straws and/or vapor phase storage tanks should be used for cryopreservation of samples from COVID-19-positive patients [157].

## 6. Conclusions

The organ damage associated with SARS-CoV-2 is caused by direct viral invasion, by the cytopathic effect of the virus, as well as by the downregulation of ACE2 and the activation of the ACE/Ang II/AT1R pro-inflammatory cascade [13]. In fact, the activation of the RAS triggers a robust innate immune response that is the probably the main cause of the clinical features of COVID-19.

RAS is highly expressed in the lungs, which explains the classical manifestation of COVID-19 as related to the respiratory system. Nonetheless, genomic and proteomic studies show the presence of local RAS in other districts, including the testes. Therefore, the possible effect of SARS-CoV-2 infection on these organs need to be clarified [43,44,45].

Concerns about male fertility started to arise when epidemiologic studies on the gender distribution observed that male patients are more affected and with increased severity by SARS-CoV-2 infection. The hormone-regulated expression of ACE2 and other RAS receptors seems to be the main reason for this discrepancy [32].

Bioinformatic studies reported the abundant expression of the ACE2 receptor and TMPRSS2 in the male reproductive tissues and cells (spermatogonial stem cells, spermatids and spermatozoa), suggesting the particular vulnerability of the testis to the virus [34]. The testicular RAS seems to have an important role in puberty onset, spermatogenesis, endocrine function and sperm capacitation, as well having the ability to affect the fluid homeostasis of the testes [54,55,68]. Nevertheless, at present, we do not have clues on how the activation of AT1R vs. AT2R by Ang II contributes to the infective and inflammatory conditions, nor to other consequences triggered by these molecules [164].

Some recent studies were able to find viral RNA in the seminal fluid of affected patients [143] and orchitis was associated with COVID-19 [144]. It is still unclear whether the putative damage to the testes is immune-mediated or caused by the viral entry, depending on the severity of symptoms and a temporary high viral load during the acute phase of the disease.

In addition, the therapeutic approaches adopted to fight the infection may have collateral toxicity that further suggests the need for a follow-up on testicular functionality in recovered patients. No data on fertility are available on Tocilizumab and Remdesivir, the most promising drugs for the fight against SARS-CoV-2, whereas the continued use of chloroquine might impair sperm quality [165]. Evidence is still insufficient to support conclusions on this topic, and follow-up studies examining the long-term effects are needed.

The effect of the infection on sperm quality is still to be clarified, as well as the virus’s presence and the infectivity of sperm samples. For ART procedures, it would be helpful to understand if semen washing by density gradient centrifugation or by a “direct swim-up” technique clears the semen from the virus, making its use in IVF cycles safe. In particular, laboratory guidelines are needed to undertake urgent semen procedures, such as cryopreservation in oncology patients.

As recommended by all the reproductive medicine associations worldwide, data relating to the potential infectivity of seminal fluid do not exclude risks for both personnel and patients. Patient risk evaluations should be carried out, good laboratory practices should be strictly followed and extra care should be taken to reduce exposure to follicular fluid and sperm, by the dilution and safe disposal of fluids.

Future studies on fertility and SARS-CoV-2 in male patients should be strongly considered, and further investigation on ACE2’s role in the gonads should be carried out for the proper management of fertility issues and in vitro fertilization.

## Figures and Tables

**Table 1:** Renin–angiotensin system component localization in the male reproductive system.

LOCATION		ACE [44,57,58]		ACE2 [21,59]	AT1R [55,60,61]	AT2R [55,61,62]	MAS [53,63]	TMPRSS2 [64,65,66,67]	REFERENCES
		sACE	tACE						
	PROSTATE	X			X	X		X	[44,55,57,58,60,61,62,64,65,66,67,68]
	VAS DEFERENS				X				[55,60,61]
	EPIDIDYMIS	X			X	X		X	[44,55,57,58,60,61,62,64,65,66,67,68]
	SERTOLI CELLS			X			X		[53,59,63,67]
	LEYDIG CELLS	X		X	X		X	X	[44,53,55,57,58,59,60,61,63,64,65,66,67]
	SEMINAL PLASMA	X						X	[44,57,58,64,65,66,67]
	SPERMATOGONIA			X			X	X	[53,59,63,64,65,66,67]
	SPERMATOCYTES						X		[53,63]
	SPERMATIDS		X		X		X	X	[44,53,55,57,58,60,61,63,64,65,66,67]
S P E R M A T O Z O A	ACROSOMAL REGION		X				X		[44,53,57,58,63]
	EQUATORIAL SEGMENT		X			X			[44,55,57,58,61,62]
	POST ACROSOMAL REGION		X			X			[44,55,57,58,61,62]
	MID PIECE		X						[44,57,58]
	FLAGELLUM		X				X		[44,53,57,58,63]
